# Non-Hodgkin Lymphoma With Longitudinally Extensive Transverse Myelopathy as the Initial Symptom: A Case Report

**DOI:** 10.3389/fonc.2019.00266

**Published:** 2019-04-12

**Authors:** Yanxing Chen, Caixiu Lin, Baorong Zhang

**Affiliations:** Department of Neurology, The Second Affiliated Hospital, School of Medicine, Zhejiang University, Hangzhou, China

**Keywords:** lymphoma, LETM, encephalitis, demyelinating diseases, CNS involvement

## Abstract

Identifying lymphoma as the cause of neurological disease is diagnostically challenging when the clinical manifestations are atypical. We report an unusual case of a previously healthy immunocompetent 67-years-old man presenting with acute onset of symptoms of myelopathy and mild personality changes. Magnetic resonance imaging (MRI) revealed multifocal periventricular lesions and longitudinally extensive transverse myelitis (LETM). He had very good response to corticosteroids and was in remission for over 6 months. Repeat MRI showed an unexpected mass lesion in the brain which was later confirmed by brain biopsy as diffuse large B cell lymphoma. Subsequent FDG-PET/CT revealed systemic disease with lymphonodal and testicular manifestations (Stage IV disease). It is therefore important to consider lymphoma as a differential diagnosis in patients with LETM and demyelinating lesions in the brain.

## Background

Diffuse large B cell lymphoma (DLBCL) is the most common type of non-Hodgkin lymphoma (NHL) worldwide and represents almost 30–40% of all lymphomas (1). The involvement of the central nervous system (CNS) is a relatively uncommon manifestation and usually indicates devastating and fatal consequences (2). Inflammatory demyelinating disorders of the central nervous system like acute disseminated encephalomyelitis (ADEM) or neuromyelitis optica spectrum disorder (NMOSD) can manifest as multifocal neurological symptoms, such as myelitis and behavioral changes, with multiple cerebral lesions, and longitudinally extensive transverse myelopathy (LETM) on MRI. Here, we report an unusual case of DLBCL presenting with an acute myelopathy and mild personality changes as the initial symptoms. LETM and multifocal periventricular lesions, particularly adjacent to the temporal horn, were seen on MRI. The diagnosis of DLBCL with CNS involvement was eventually confirmed by brain biopsy after the 6-month follow-up.

## Case Presentation

A previously healthy 67-year-old man presented with an acute onset of back pain, followed by progressive numbness and weakness of the limbs for 1 week. He could still ambulate with minimal help. On the day he was admitted to our hospital, his condition aggravated suddenly and reached a plateau within 8 h. He developed urinary retention and could barely raise his arms or move his legs. His family members mentioned that he also became irritable and forgetful. The neurological examination revealed severe quadriparesis (Medical Research Council strength score, upper extremities: grade 3; lower extremities: grade 1), a sensory C4 level, tendon hyperreflexia, and Hoffmann and Babinski sign. Physical examination was unremarkable.

**Figure 1:**
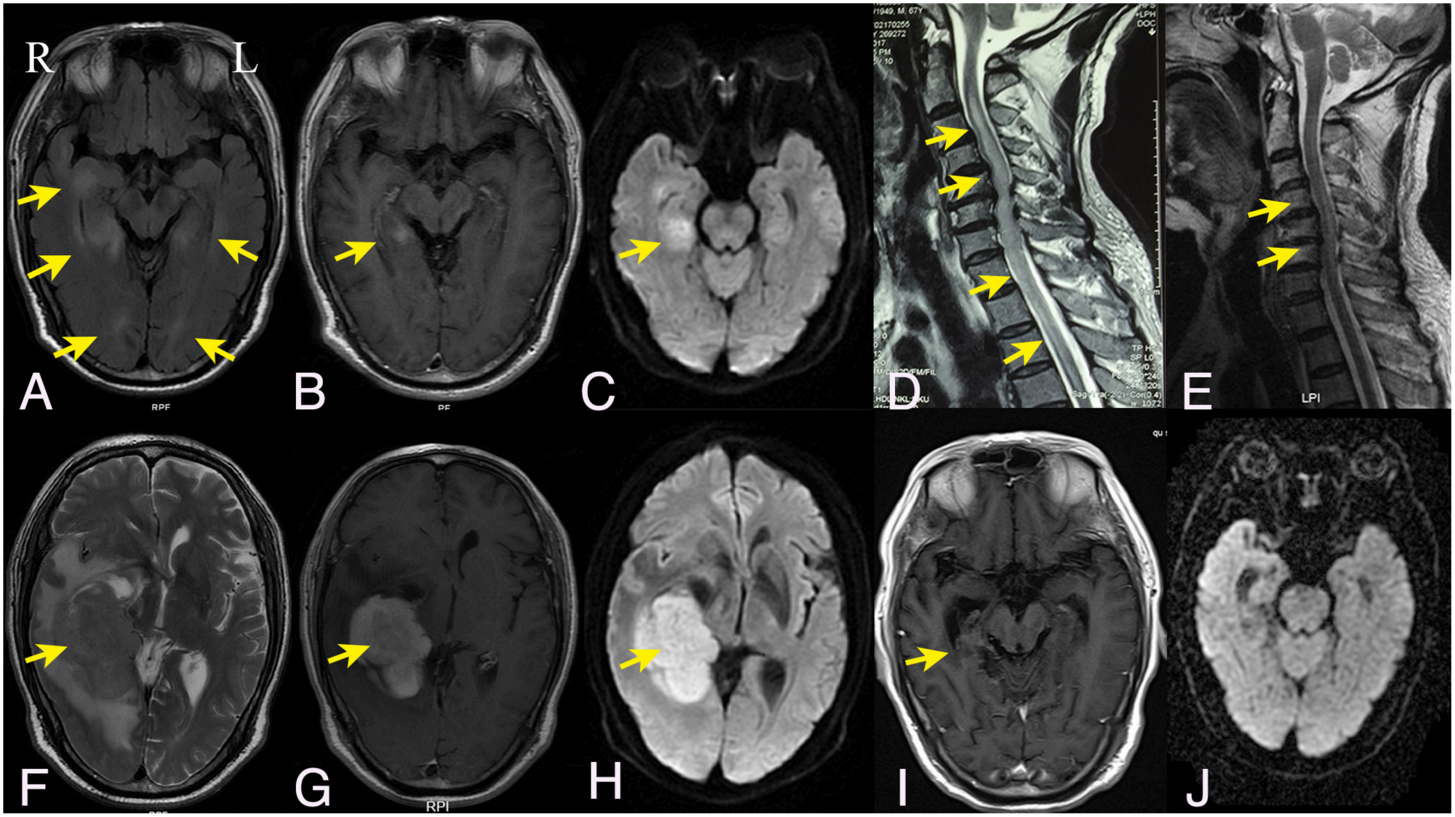
Brain and spine MRI findings. The initial axial fluid-attenuated inversion recovery **(A)**, axial T1-weighted gadolinium-enhanced **(B)**, and axial diffusion weighted imaging **(C)** brain MRI showed bilateral irregular hyperintense lesions around the ventricles with enhancement on the right temporal lobe. **(D)** Sagittal T2-weighted cervical spine MRI showed hyperintensity longitudinally extending from the cervical to the thoracic cord. **(F–H)** Six months later, the follow-up axial T2-weighted brain MRI **(F)** and diffusion weighed imaging **(H)** showed a mass lesion in the right temporal lobe with homogenous enhancement on T1-weighted gadolinium-enhanced series **(G)**; Sagittal T2-weighted cervical spine MRI showed regredient lesions in the cervical cord **(E)**. **(I,J)** After the chemotherapy, the right temporal lesion on T1-weighted gadolinium-enhanced series **(I)** was significantly regredient and necrotic; No abnormal signal was displayed on diffusion weighted imaging **(J)**. R indicates right and L indicates left.

Laboratory investigations including full blood counts, thyroid function, liver and renal function, LDH, β2-microglobulin, and tumor markers were normal. HIV antibodies and syphilis antibodies were negative. Antibodies mediating autoimmune encephalitis (anti-NMDAR, -AMPAR, -LG1, -CASPR2, -GABA_B_R) or paraneoplastic syndromes (anti-Yo, -Hu, -Ri, -CV2, -amphiphysin, -PNMA2) in the cerebrospinal fluid (CSF) and serum were negative, so were antibodies against aquaporin-4 (AQP-4), myelin oligodendrocyte glycoprotein (MOG), and myelin basic protein (MBP). CSF analysis revealed normal CSF pressure, a mild pleocytosis (8 cells/ml) without atypical or malignant cells, an elevated protein level (132.9 mg/dl), and normal glucose and chlorine levels. Bacterial and fungal cultures were negative. CSF IgG index was 0.17, and the IgG oligoclonal bands were absent. Brain MRI revealed multifocal periventricular lesions with gadolinium enhancement in the left medial temporal lobe (Figures 1A–C). Spine MRI revealed longitudinally extensive abnormal signal extending from the cervical to the thoracic cord (Figure 1D).

The patient was suspected to have inflammatory demyelinating disease with a working diagnosis of ADEM or serologically negative NMOSD. He was treated with intravenous methylprednisolone (1 g/d for 5 days), followed by prednisone taper, and his condition markedly improved. The back pain was substantially relieved. Neurological examination revealed improved extremity strength (upper extremities: grade 4+; lower extremities: grade 4), with a sensory level of T12. He was subsequently treated with intravenous immunoglobulin, but without further improvement. The patient was discharged to rehabilitation.

**Figure 2:**
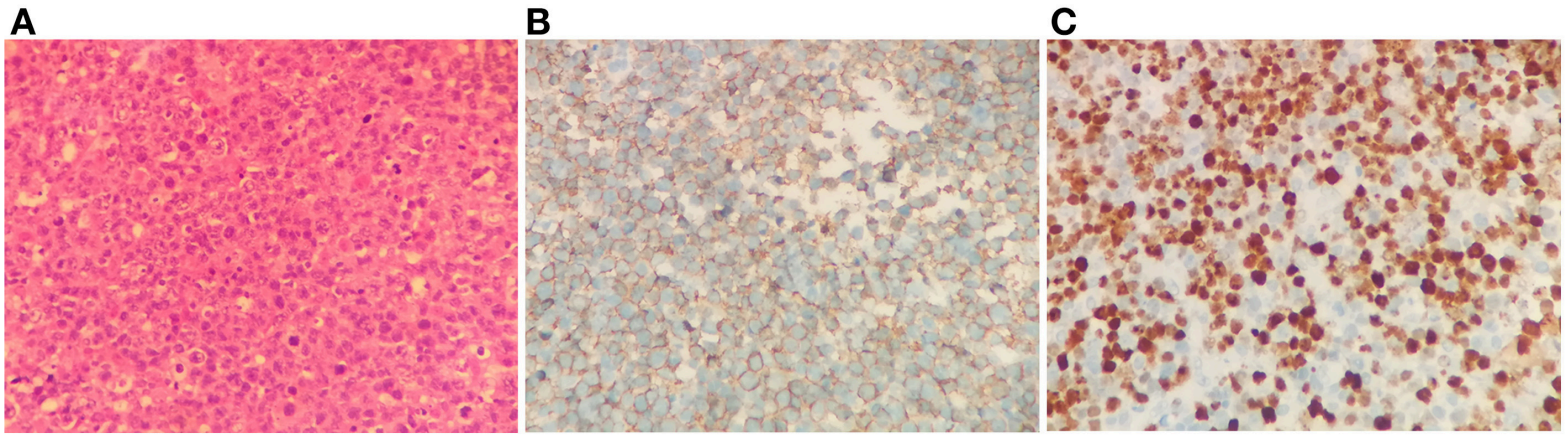
Brain biopsy findings. Hematoxylin and eosin staining revealed diffusely distributed atypical lymphoid cells **(A)**. Immunohistochemistry study revealed strong positive staining of the lymphoma cells for CD20 B cell marker **(B)**, and the Ki-67 index was 70% **(C)**. Magnification, ×400 for all.

**Figure 3:**
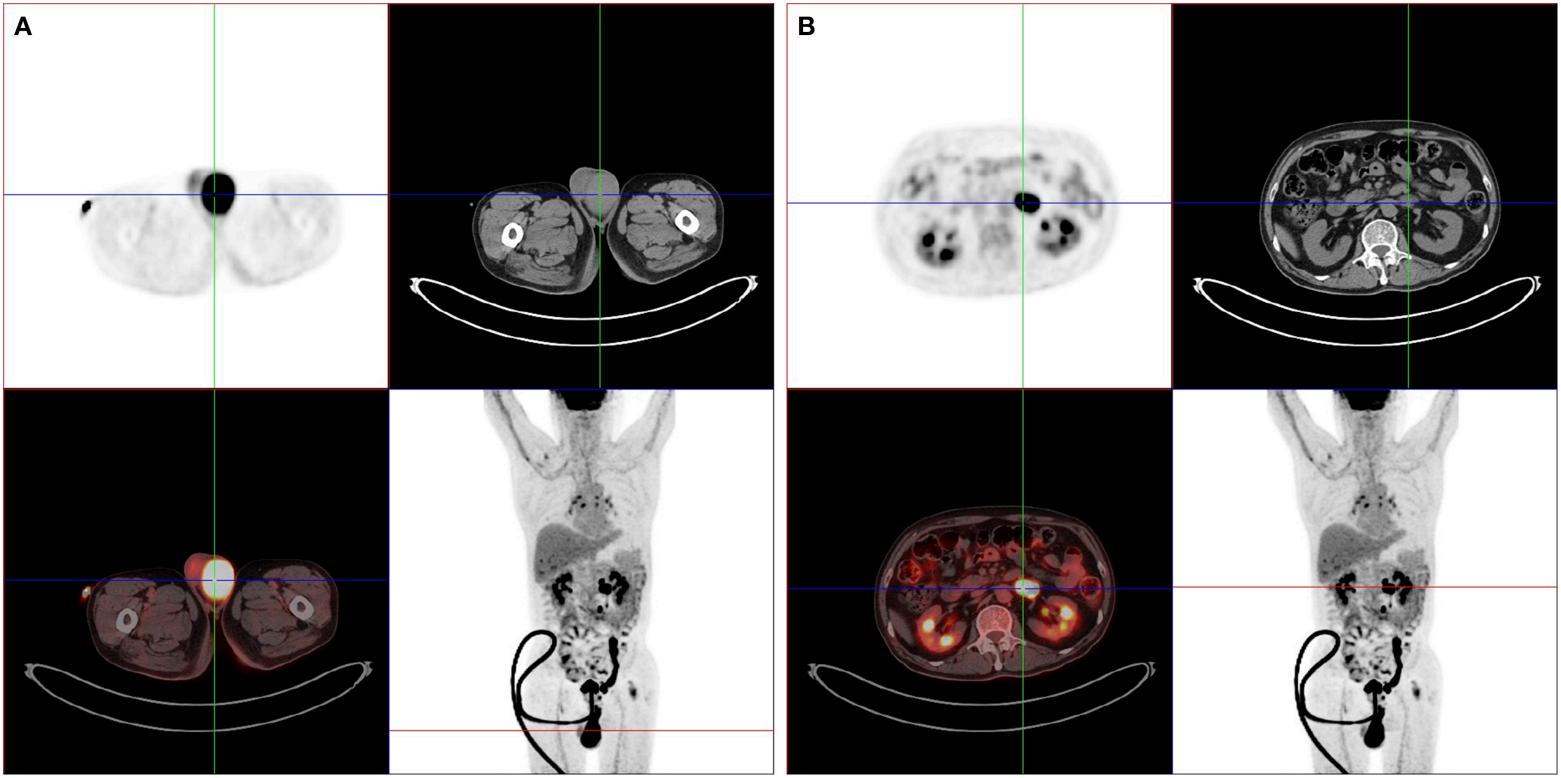
Whole-body PET/CT findings. PET/CT imagings showed abnormal hypermetabolic lesions in the left testicle **(A)** and in multiple abdominal and retroperitoneal lymph nodes **(B)**.

For the following 6 months, the patient's condition remained stable. He could ambulate with minimal help. However, the 6-month follow-up brain MRI revealed an unexpected enhancing temporal mass with extensive perifocal edema (Figures 1F–H). Repeat spine MRI revealed decreasing lesions without contrast enhancement (Figure 1E). Brain stereotactic biopsy of the right temporal mass was performed. Histopathological findings of the specimen demonstrated DLBCL (Figure 2). Whole-body positron-emission tomography/computed tomography (PET/CT) imaging was performed to evaluate extra-neural involvement. Abnormal hypermetabolism was found in the left testicle and in multiple abdominal and retroperitoneal lymph nodes (Figure 3). Subsequent testicle and bone marrow biopsies were consistent with DLBCL (Stage IV disease).

Systemic chemotherapy (Methotrexate + Rituximab + Ara-C) combined with involved field radiotherapy was conducted. Due to severe vomiting, the chemotherapy had to be discontinued after the fourth therapy cycle. Repeat brain MRI showed necrosis of the original mass lesion, no new lesions were observed (Figures 1I,J). Sixteen months after the initial admission, he could ambulate by himself without any help.

## Discussion and Conclusions

DLBCL, the most common type of NHL, occurs primarily in middle-aged or elderly patients. The median age at the time of diagnosis is in the 7th decade (1). Gastrointestinal tract is the most common site of lymphoma. NHL confined to the brain, leptomeninges, spinal cord, or eyes without evidence of systemic involvement, is called primary CNS lymphoma. Primary CNS lymphoma is rare and aggressive, accounting for 1% of all lymphomas (3). If the spinal cord is involved, primary CNS lymphoma is often found to have occult systemic involvement (4). Therefore, it is very rare for systemic NHL to present with acute extensive myelitis as the initial manifestation, as it was the case in this report.

Single mass lesion is the most common manifestation of CNS lymphoma (3). It can also occur with a variable and atypical imaging appearance, rendering the diagnosis unusually challenging. The brain lesions of our patient were diffuse without mass effect or edema, and were located mainly in the medial temporal lobe. The patient presented with acute onset of mild personality changes, leading us to suspect presence of autoimmune encephalitis. Spinal cord involvement has been reported in autoimmune encephalitis, though is considered to be very rare. Extensive myelitis has been reported to be associated with anti-NMDA receptor antibodies (5, 6). The failure to detect autoimmune antibodies in the CSF or serum prompted us to consider other differential diagnoses.

The clinical features of CNS lymphoma are often of subacute onset and are variable depending on the site of the CNS involved. This patient presented with acute back pain, followed by quadriparesis. The spine MRI revealed longitudinally extensive transverse T2 hyperintensity. LETM is not rare but is usually associated with inflammatory or demyelinating diseases. In cases of LETM, lymphoma is rarely considered as a differential. Taking the brain lesions and acute onset of manifestation into account, we still considered that inflammatory demyelinating disorders such as ADEM or NMOSD would be the most probable diagnoses, although the antibodies against AQP-4, MOG, and MBP were negative, so were CSF oligoclonal bands. Given the severity of the symptoms, we treated the patient with corticosteroids, which turned out to be very effective in improving the acute symptoms.

The follow-up spine MRI showed significantly improved lesions without contrast enhancement or PET/CT hypermetabolism. Therefore, the longitudinally transverse myelitis might have been paraneoplastic, secondary to diffuse large B cell lymphoma, or, less probable, might as well have been a DCBCL manifestation promptly responding to corticosteroids. Primary CNS lymphoma can mimic other brain disorders including inflammatory demyelinating diseases, and in rare cases can be misdiagnosed as multiple sclerosis (MS), ADEM, transverse myelitis, or NMOSD. Initial presentation as primary intramedullary myelopathy has been documented to delay a definitive diagnosis of lymphoma by a median of 8 months in a series of 14 subsequent patients at Mayo Clinic (7). As a matter of fact, CNS lymphoma can be preceded by sentinel demyelinating lesions. It has been reported that histologically confirmed inflammatory demyelinative lesions turn out to be diffuse large B cell lymphoma several months later by repeated brain biopsy (8, 9). As in this patient, no spinal biopsy has been performed, follow-up studies will have to include MRI of the spine to cover up for a possible, spinal manifestation of DLBCL.

Atypical brain lesions or LETM, though rare, have been reported in cases which were later diagnosed as CNS lymphoma. But only one case reported in 2018 presented with both brain lesions and LETM (10). That patient had a gradual onset of symptoms over 8 months, which initially improved with the treatment of corticosteroids, but soon deteriorated to bed bound within 1 month. In the current case, the patient presented with quite different features, including acute onset, good response to corticosteroids with sustained symptom relief for over 6 months until the follow-up evaluation.

In conclusion, we report a case of systemic NHL with acute onset of LETM-like presentation as the initial symptoms. The clinical symptoms and the imaging characteristics were very sparse, resembling diseases like auto-immune encephalitis or inflammatory demyelinating diseases, and showed very good response to corticosteroids. It is, therefore, important to closely follow-up in patients suspected of demyelinating disorders without a definitive diagnosis, even in case of very good initial response to treatment.

## Ethics Statement

The study was approved by the Human Ethics Review Committee of the Second Affiliated Hospital, Zhejiang University.

## Consent to Publish

A written informed consent to publish the report and associated medical images was obtained from the patient.

## Author Contributions

YC and CL analyzed the data, drafted, and revised the manuscript. BZ designed the study and revised the manuscript. All authors of this manuscript have actively participated in the data acquisition, and they all commented and approved the final version of the manuscript.

### Conflict of Interest Statement

The authors declare that the research was conducted in the absence of any commercial or financial relationships that could be construed as a potential conflict of interest.
